# Current trends of clinical trials involving CRISPR/Cas systems

**DOI:** 10.3389/fmed.2023.1292452

**Published:** 2023-11-10

**Authors:** Songyang Zhang, Yidi Wang, Dezhi Mao, Yue Wang, Hong Zhang, Yihan Pan, Yuezeng Wang, Shuzhi Teng, Ping Huang

**Affiliations:** ^1^The Key Laboratory of Pathobiology, Ministry of Education, Norman Bethune College of Medicine, Jilin University, Changchun, China; ^2^The Third Affiliated Hospital of Jilin University, Changchun, China; ^3^The Second Affiliated Hospital of Jilin University, Changchun, China

**Keywords:** CRISPR, genome editing, clinical trials, cancer, hemoglobinopathies, COVID-19, HIV, gene therapy

## Abstract

The CRISPR/Cas9 system is a powerful genome editing tool that has made enormous impacts on next-generation molecular diagnostics and therapeutics, especially for genetic disorders that traditional therapies cannot cure. Currently, CRISPR-based gene editing is widely applied in basic, preclinical, and clinical studies. In this review, we attempt to identify trends in clinical studies involving CRISPR techniques to gain insights into the improvement and contribution of CRISPR/Cas technologies compared to traditional modified modalities. The review of clinical trials is focused on the applications of the CRISPR/Cas systems in the treatment of cancer, hematological, endocrine, and immune system diseases, as well as in diagnostics. The scientific basis underlined is analyzed. In addition, the challenges of CRISPR application in disease therapies and recent advances that expand and improve CRISPR applications in precision medicine are discussed.

## 1. Introduction

### 1.1. The principles of the bacterial CRISPR/Cas9 system

**Figure 1:**
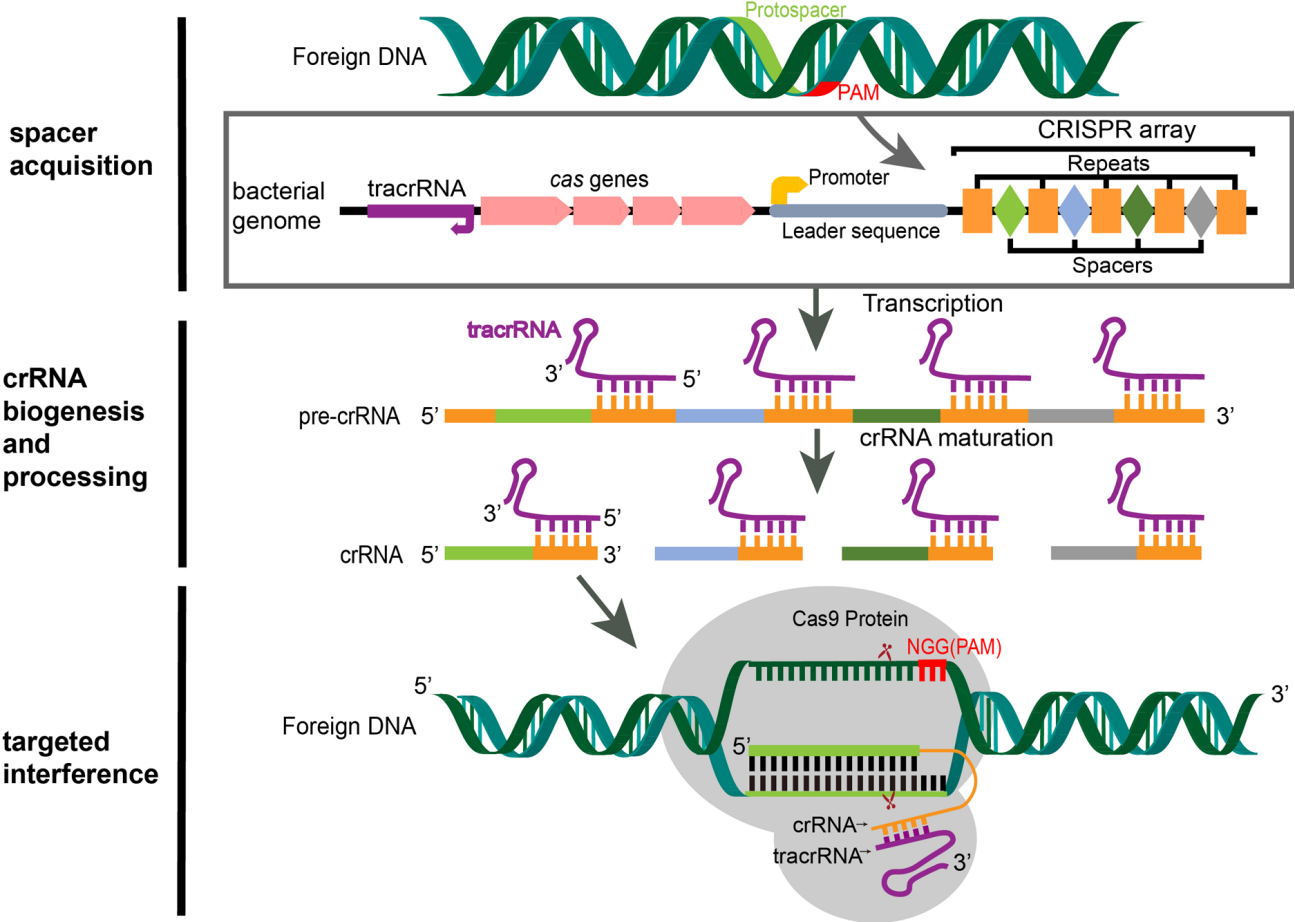
CRISPR/Cas9 mediated adaptive immunity in bacteria. A typical CRISPR locus consists of a leader sequence followed by an array of short identical repeats interspaced by short unique spacer sequences, as well as a set of CRISPR-associated (*cas*) genes (5). Preceding the *cas* genes is the tracrRNA, which encodes a non-coding RNA that is complementary to the repeats. During the acquisition stage, foreign DNA was cleaved into short DNA fragments (protospacers) and incorporated into the CRISPR array in chronological order of invasion as a spacer (6). Once integrated, the new spacer is transcribed with all other spacers into a pre-crRNA. The tracrRNA is transcribed separately and combines with the repeat sequence of the pre-crRNA to form a heterodimer. Then, the heterodimer RNA is cut by RNase III to form mature crRNA (7). When the same foreign DNA invades again, the mature crRNA-tracrRNA structure engages the Cas9 protein to form an RNP. RNP guides the Cas9 protein to recognize the PAM sequence (NGG for SpCas9) of the foreign DNA by matching the crRNA with the exogenous genes and performing site-specific double-strand cleavage at the three bases upstream, then the foreign DNA sequence is destroyed (6).

The CRISPR/Cas (clustered regularly interspaced short palindromic repeats/CRISPR-associated) endonuclease system is an acquired immune system of bacteria and archaea formed during their long-term evolutions to provide them with resistance to exogenous viruses or plasmids (1–4). The CRISPR locus is composed of a CRISPR array and an upstream *cas* operon that contains all the Cas protein-coding genes (Figure 1). The CRISPR array is an alternating “repeat-interval-repeat” sequence first found in the bacterial genome. The direct repeat sequences were found only existing in bacteria and archaea but not in viruses and eukaryotes (8) and identified as a group of identical sequences containing 29 nucleotides (nt), which were separated by 32-nt spacer sequences (9), originated from the invading bacteriophage genomes or conjugative plasmids. These bacteria and archaea would not be reinfected by the phages or plasmids containing the same spacer sequence (10). In 2007, Barrangou et al. confirmed that the CRISPR/Cas system is indeed a bacterial acquired immune system and that the CRISPR spacer sequences confer resistance to specific bacteriophages (11).

Based on the diversity of *cas* genes and CRISPR effector complexes, the CRISPR/Cas system is categorized into 2 classes, 6 types, and at least 33 subtypes (12, 13). Class 1 possesses multi-subunit effector complexes and is subdivided into Types I, III, and IV, while Class 2 has a single protein effector and is subdivided into Types II (Cas1, Cas2, and Cas9), V (Cas12), and VI (Cas13). In bacteria, CRISPR/Cas immune systems operate through three consecutive steps: (1) the acquisition of exogenous DNA; (2) the biosynthesis of CRISPR RNA (crRNA); and (3) target interference (14) (Figure 1).

In the first step, invasive exogenous DNA is cleaved into short DNA fragments, called protospacers, by Cas nuclease complexes in bacteria, and the protospacers are integrated into the CRISPR locus in chronological order of invasion and separated by repeats. There is a 2–5 bp protospacer-adjacent motif (PAM) sequence at the 3′ end of the foreign DNA protospacer, which does not integrate into the host genome (6, 15) and therefore can be used as a marker to distinguish the host genome from foreign sequences (16), preventing bacterial self-cleavage by Cas9 during targeted interference (17, 18). During the biosynthesis of crRNA, the CRISPR array sequences are transcribed into a long precursor crRNA (pre-crRNA). The upstream trans-activating crRNA (tracrRNA) is transcribed separately and complements the repeat sequence of the pre-crRNA to form a heterodimer, which is cut by RNase III to form a mature crRNA that contains a complete spacer sequence and a partial repeat sequence (7). Cas9 plays a role in stabilizing the pre-crRNA-tracrRNA complex in this process. The third step is targeted interference. When the same foreign DNA invades again, the mature crRNA and tracrRNA combine to form guide RNA (gRNA), which binds to the Cas9 protein to form a ribonucleoprotein (RNP). The RNP specifically recognizes the protospacer on the invading foreign DNA and guides the Cas9 endonuclease to perform site-specific double-stranded DNA (dsDNA) cleavage at the three bases upstream of PAM; thus, the foreign DNA is destroyed and ultimately eliminated (6).

### 1.2. The adaptation of the CRISPR/Cas9 system in mammalian cells

In 2012, Jinek and colleagues constructed a chimeric single guide RNA (sgRNA) by fusing the 3′ end of crRNA to the 5′ end of tracrRNA, and the combination is sufficient to guide Cas9-mediated dsDNA cleavage (6). This work established the first CRISPR/Cas9 genome editing tool, which has been successfully applied to mammalian cells (2, 15). The CRISPR/Cas9 system mediates precise dsDNA breaks at the target site to accomplish genome editing through primarily two pathways: the major one is non-homologous end joining (NHEJ), which occurs in all phases of the cell cycle, involves the direct ligation of blunt or sticky ends, and often results in small insertions and deletions (indels) that generate frame-shift mutations or premature stop codons at the cut site; and the other one is homology-directed repair (HDR), which uses a homologous template for DNA repair, is restricted to the S/G2 phase of the cell cycle, and has higher fidelity but is less efficient than NHEJ (19). Targeted mutations can be introduced if the specific mutations exist in the template DNA (20).

The Cas9 of *Streptococcus pyogenes* (SpCas9) has been adapted for RNA-guided single or multiple genome editing (2, 15), gene activation, and suppression in a variety of organisms in the presence of different crRNA but can mediate only a single activity at a time within any given cell (21). Cas9 orthologs from distinct bacterial species have been identified, e.g., *Staphylococcus aureus* Cas9 (SaCas9), and coexpression of Cas9 variants can mediate concomitant and independent targeted gene regulation and editing in bacteria and human cells in the presence of paired crRNA and tracrRNA (21). CRISPR/Cas9 mediates precise cleavage of endogenous DNA and induces multiplex editing of target loci, indicating the programmability and wide applicability of this technology at the genomic level (2, 15). Of note, the first-in-human clinical trial using CRISPR/Cas9 technology was carried out in 2016 to treat non-small cell lung cancer (NSCLC) (22).

### 1.3. The development of CRISPR/Cas systems

SpCas9 recognizes the PAM sequence of NGG, which limits the ability of the CRISPR system to make a precise cut in many hereditary diseases. Cas9 orthologs and more CRISPR Cas endonucleases with paired PAMs are in great need. The newly evolved base editors (BEs) (23) and prime editors (PEs) (24) provide powerful tools for single nucleotide conversion without inducing DNA double-strand breaks (DSBs), thus reducing genotoxicity.

#### 1.3.1. CRISPR/Cas9 orthologs and other CRISPR/Cas systems

The canonical PAM sequence 5’-NGG-3′ is associated with SpCas9. Different PAMs have been found to be associated with the bacterial Cas9 proteins of *Streptococcus thermophiles* (St1Cas9) (25), *Neisseria meningitides* (Nm1Cas9) (26), *Treponema denticol* (TdCas9), and *Francisella novicida* (FnCas9) (27). These Cas9 orthologs and their engineered variants recognize different PAM sites at any desired genome location for precise editing with altered and improved PAM specificities (27), leading to an increased number of diseases that could be treated via gene editing.

Novel CRISPR/Cas systems have been identified as well. Cas12a (Cpf1, CRISPR from *Prevotella and Francisella* 1) is a small single RNA-mediated endonuclease independent of tracrRNA (28) and provides a T-rich PAM recognition site (TTTV, V = A/C/G) for genome editing and generates sticky ends that are more effective for precise DNA insertion. CRISPR/Cas13a (C2c2) from the bacterium *Leptotrichia shahii* is characterized as an RNA-guided CRISPR system that targets RNA but not DNA sequences (29). Aside from the CRISPR/Cas systems mentioned above, novel Cas endonucleases with paired PAMs and their engineered variants are yet to come.

#### 1.3.2. Base editing

The base editing system fuses a deaminase domain to a Cas9 nickase (nCas9) to convert an A·T to a G·C base pair (catalyzed by adenine base editors, ABEs) or a C·G to a T·A base pair (catalyzed by cytosine base editors, CBEs) without the need for donor templates (23). With the emergence of new generations of BEs, their editing efficiency and applicability have been greatly improved (30). ABE8s were generated with higher editing efficiency and an expanded editing window to A3–A10 in NGG PAM (at positions 21–23) (31). The broadened editing window enables the precise correction of 4,724 (23.3%) pathogenic G > A or C > T mutations identified in the ClinVar database (31). The cytosine base editors BE4max and AncBE4max were created through modification of nuclear localization signals, ancestral reconstruction of the deaminase component, and codon usage, which showed increased editing than former generations of CBEs (32). Collectively, ABEs and CBEs enable the correction of 61% of human pathogenic SNPs in the ClinVar database (32).

Recently, glycosylase base editors (GBEs) were developed to induce C-to-A and C-to-G transversion in *Escherichia coli* and mammalian cells, respectively (33, 34). Besides, Tong et al. constructed an adenine transversion base editor (AYBE, Y = C or T) for effective A-to-C and A-to-T editing by fusing an ABE with hypoxanthine excision protein N-methylpurine DNA glycosylase (35). Altogether, these base editors can make all types of base transitions and transversions.

#### 1.3.3. Prime editing

In 2019, David Liu’s group developed a prime editing system that enables all kinds of nucleotide conversion, insertion up to 44 bp, and deletion up to 80 bp in a controlled manner without DSBs or an exogenous DNA template (24). The first generation of prime editor (PE1) consists of nCas9 (H840A) fused to an engineered reverse transcriptase (RT) and prime editing guide RNA (pegRNA), which contains sgRNA, RT template, and PBS (primer binding site). Guided by pegRNA, nCas9 produces a single-strand break at the PAM-containing strand to expose a 3′ flap that hybridizes with the PBS of pegRNA, and the 3′ flap was extended by reverse transcriptase using the RT template containing the desired edit. During the cellular DNA repair process, the editing outcome depends on which flap is degraded. If the original 5′ flap at the cleavage site is degraded, the 3′ flap with the edited sequence is incorporated into the PAM strand, and then the desired edit is introduced to the non-PAM strand by DNA mismatch repair (MMR). While 3′ flap degradation restores the original unchanged sequence. Subsequently, Liu’s group developed PE2 containing an RT variant with five amino acid substitutions and PE3 using an additional sgRNA to induce a second nick on the non-PAM strand to enhance prime editing efficiency (24). Furthermore, PE with engineered pegRNA (epegRNAs) (36), bi-direction PE (37), split PE (38), and stem-loop PE (39) have been produced to increase prime editing efficiency and precision and to be applied in a variety of settings.

Since CRISPR/Cas gene editing technology is highly efficient, cost-effective, and easy to use, it has been widely applied to basic and translational research, as well as in the diagnosis and treatment of human diseases. This review summarizes both completed and ongoing CRISPR-related clinical trials, of which over 80% use CRISPR/Cas9, with specific emphasis on several types of diseases for which CRISPR-based technologies have been widely used. Moreover, current challenges, potential solutions, and the future perspective of the employment of CRISPR/Cas platforms in clinical settings are also discussed.

## 2. Database searching and screening for CRISPR clinical trials

**Figure 2:**
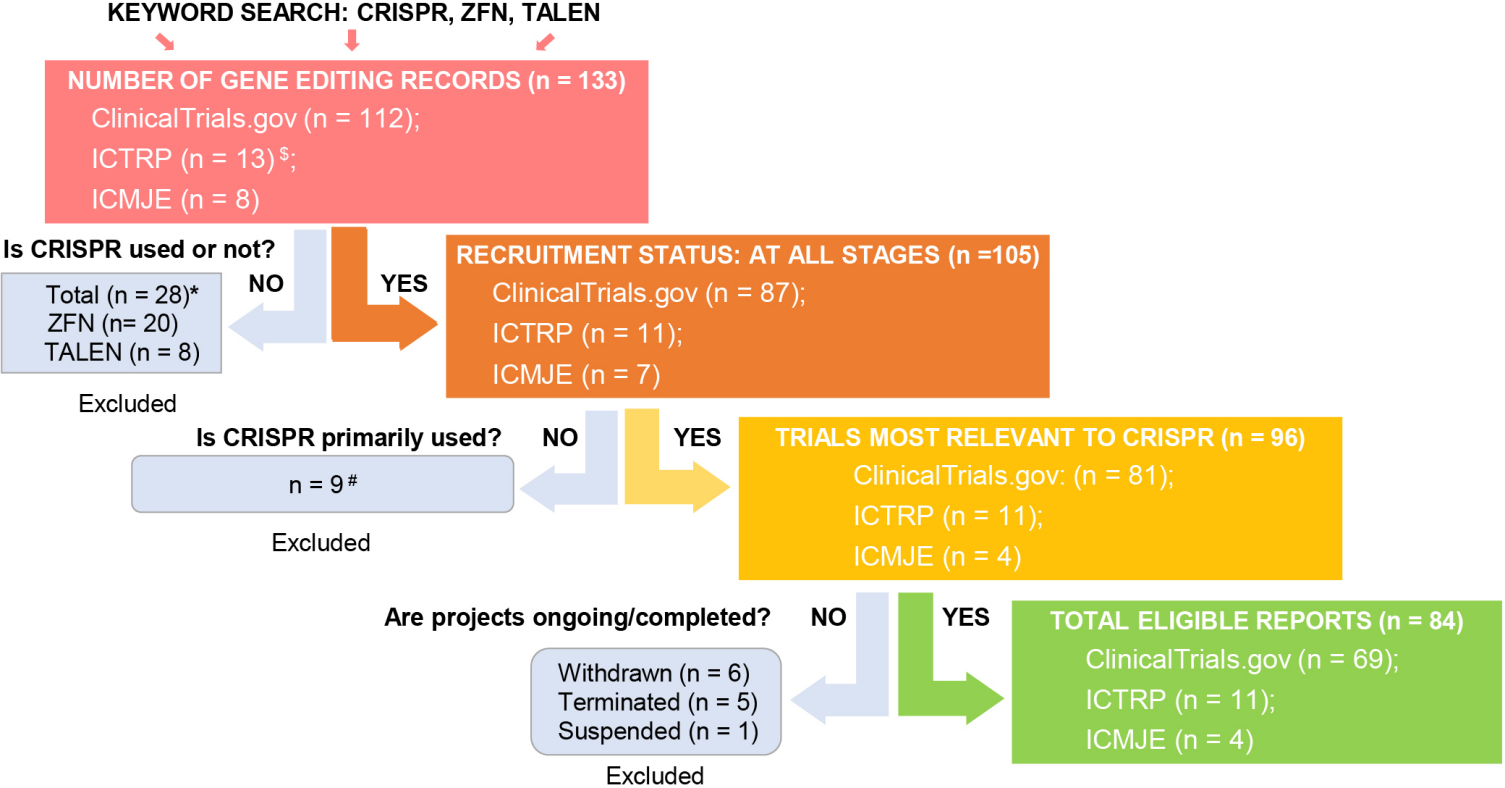
Screening for global CRISPR clinical trials. We assessed clinical trials registered at ClinicalTrials.gov, ICTRP, and ICMJE. ^$^Fifty-two and two duplicate CRISPR clinical trials were found in ICTRP and ICMJE, respectively, and were removed. *There were 20 records using ZFN (clinicaltrials.gov: 17, ICTRP: 2, ICMJE: 1) and 8 records using TALEN (clinicaltrials.gov: 8, ICTRP: 0, ICMJE: 0). ^#^Basic study records and those not using CRISPR as a primary technique were excluded after being assessed for eligibility. Excluded trial identifiers: NCT03342547, NCT03450369, NCT04122742, NCT04478409, NCT05443607, NCT03681951, CTRI/2018/09/015807, CTRI/2021/09/036609, and CTRI/2023/09/057289.

**Figure 3:**
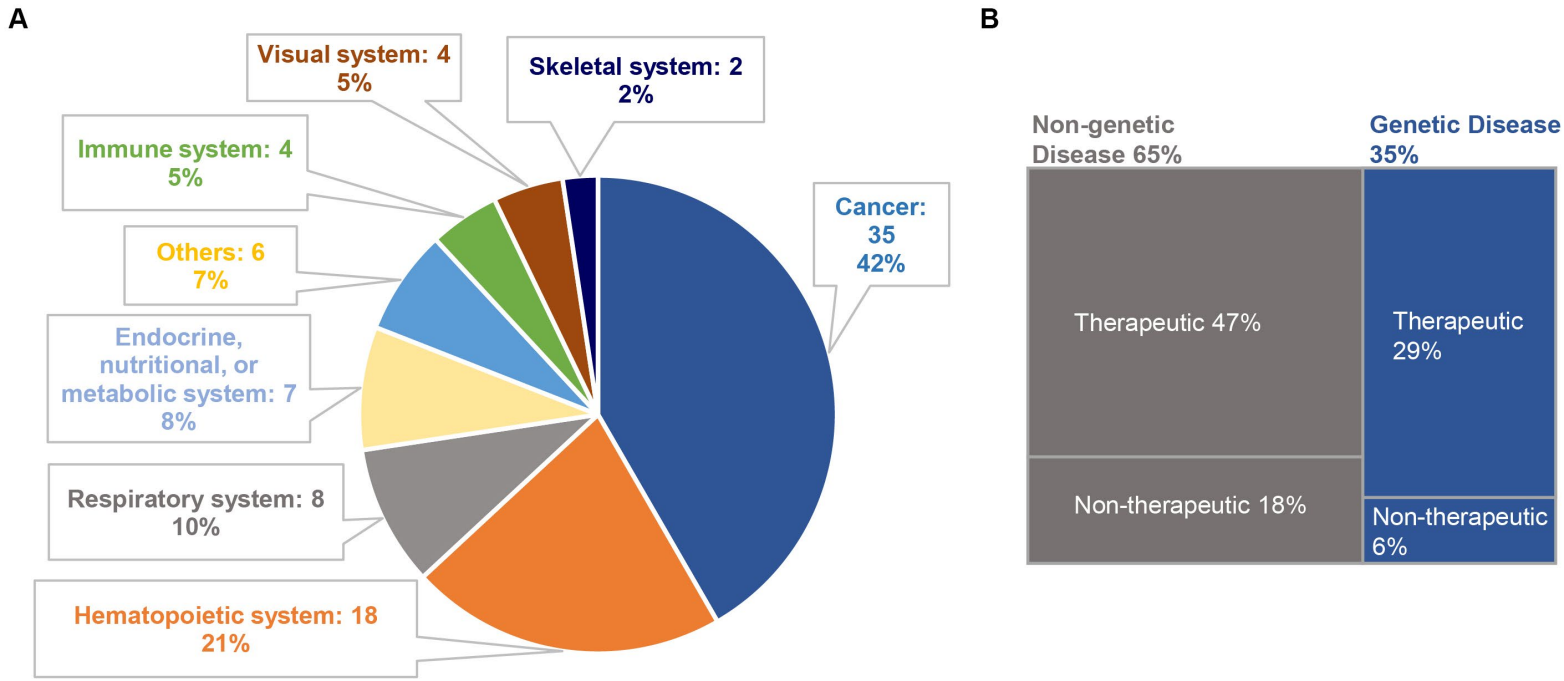
Classification of CRISPR-based clinical trials. **(A)** A total of 84 clinical trials are classified according to disease categories; **(B)** among them, 29 trials and 55 trials are related to genetic diseases (35%) and non-genetic diseases (65%), respectively. We further divide the selected clinical trials into therapeutic trials and non-therapeutic trials. In genetic diseases, 5 trials are non-therapeutic, and the remaining 24 trials are therapeutic. Among the non-genetic diseases, 15 trials are non-therapeutic, and 40 trials are therapeutic.

To evaluate the current trends in clinical trials using different gene editing technologies globally, we accessed records from multiple clinical trial registry databases at ClinicalTrials.gov, the International Clinical Trials Registry Platform (ICTRP), and the International Committee of Medical Journal Editors (ICMJE). There are 20 and 8 clinical trials using zinc finger nucleases (ZFNs) and transcriptional activator-like effector nucleases (TALENs), respectively (Figure 2). Compared to ZFNs and TALENs that recognize target sequences via protein domains, the CRISPR/Cas systems recognize target sequences via sgRNA and are easier to operate, cheaper with lower cytotoxicity, and more widely used. From ClinicalTrials.gov, we identified 87 suitable trials by retrieving the keyword “Clustered Regularly Interspaced Short Palindromic Repeats.” Additionally, we obtained 11 CRISPR trials from the ICTRP after excluding duplicate registrations on ClinicalTrials.gov and 7 nonidentical CRISPR trials from the ICMJE database, which contains 17 registries, including the Chinese Clinical Trial Registry (ChiCTR) and the Clinical Trials Registry-India (CTRI). Ultimately, a total of 105 registered CRISPR trials were further scrutinized, as shown in Figure 2. After screening, the remaining 84 clinical trials (Supplementary Table S1) were classified according to the types of disease, and 35 of them were cancer-related (42%). The second most studied system was the hematopoietic system (21%), and clinical trials were conducted on treating β-thalassemia and sickle cell disease (SCD). Eight clinical trials (10%) were focused on the respiratory system, of which 5 were about COVID-19. Endocrine, nutritional, or metabolic-related disorders were studied in 7 cases (8%). The rest of the clinical trials were conducted for HIV infection, disorders in the visual and skeletal systems, etc. (Figure 3A).

Among these CRISPR-based clinical trials, genetic disease and non-genetic disease account for 35 and 65%, respectively. We further categorized the selected clinical trials into “therapeutic trials” and “non-therapeutic trials.” The “therapeutic” studies are referred to as interventional clinical trials using CRISPR technology for disease treatment, whereas the “non-therapeutic” studies are those primarily utilizing CRISPR technology as a tool for disease screening, disease diagnosis, and to study important factors that can advance our understanding of a disease (Figure 3B).

## 3. Disease classifications of CRISPR clinical trials

The major applications of CRISPR/Cas technology in human clinical trials are discussed below to gain useful insights.

### 3.1. Cancers

As the top application, most CRISPR-based cancer-related trials are therapeutic studies in phase 1 or phase 2, of which studies against hematologic malignancies and solid tumors represent 54 and 46%, respectively (Supplementary Table S1). Since adoptive cell transfer (ACT) of tumor-reactive T lymphocytes has emerged as one of the most promising immunotherapeutic approaches against cancer, it has been widely investigated in 86% of CRISPR clinical trials in cancer. The three types of cells for ACT are chimeric antigen receptor (CAR)-T cells, T cell receptor-engineered T (TCR-T) cells, and tumor-infiltrating lymphocytes (TILs), and they can be engineered by Cas9 RNP to enhance their therapeutic potential (40, 41).

#### 3.1.1. Hematologic malignancies

CRISPR-engineered CAR-T cell therapies have been applied in 18 out of 19 CRISPR-based clinical trials for hematologic malignancies (Table 1). CAR-T cells are generated by transducing human T cells with CARs, which are genetically engineered fusion proteins comprised of a single-chain antibody fragment as an extracellular antigen recognition moiety, linked to a spacer and a transmembrane region, followed by intracellular T-cell signaling modules such as CD28-CD3ζ or 4-1BB-CD3ζ (53, 54). Once the antibody fragment of CAR recognizes and binds to a tumor antigen on the tumor cell surface, the intracellular signaling domains transmit a stimulatory signal to activate CAR-T cells. Thus, CAR-T cell therapy combines the specificity of antibody-like recognition with the cytotoxic potency of activated T cells, enabling target elimination of tumors without relying on antigen processing and presentation by the major histocompatibility complex (MHC), more specifically identified as human leukocyte antigen (HLA). Therefore, CAR-T cells have broader applications than physiological (MHC-restricted) TCR-T cells.

**Table 1:** CRISPR-based clinical trials for CAR-T cell therapy.

Trial ID	Disease	Target	Cell type	Intervention	Phase	Country	References
NCT04035434	Relapsed/refractory B-cell malignancies	TRAC and B2M	Allogeneic T cells	CTX110: CD19-directed allogeneic T cells genetically modified *ex vivo* using CRISPR-Cas9 gene editing components	1	United States Australia Canada, France Germany, Spain	(42)
EUCTR2018-003916-38-DE	Relapsed/refractory B-cell malignancies	TRAC and B2M	Allogeneic T cells	CTX110: CD19-directed allogeneic CAR-T cell therapy with dose escalation (25 × 10^6 cells/mL–85 × 10^6 cells/mL)	1	Australia Canada Germany United States	
NCT05643742	Relapsed/refractory B-cell malignancies	Regnase-1, TGFβR2, TRAC, B2M and CD70	Allogeneic T cells	CTX112: CTX110 + disruption of Regnase-1 and TGFβR2 via CRISPR/Cas9	1/2	United States	(43)
NCT03166878	Relapsed/refractory CD19+ leukemia or lymphoma	TCR and B2M	Allogeneic T cells	UCART019: Day 0, 10% of total dose; Day 1, 30% of total dose if patient is stable (no significant toxicity) from prior dose; Day2, 60% of total dose if patient is stable from prior dose	1/2	China	
NCT04037566	Relapsed/refractory CD19+ leukemia or lymphoma	HPK1	Autologous T cells	Autologous T cells engineered to target CD19 and CRISPR-based gene editing to eliminate endogenous HPK1 (XYF19 CAR-T cells)	1	China	(44)
NCT04637763	Relapsed/refractory B-cell non-Hodgkin lymphoma	TRAC and PD-1	Allogeneic T cells	CB-010: a CRISPR-edited allogeneic CAR-T cell therapy targeting CD19	1	United States	
NCT03398967	B-cell leukemia/B-cell lymphoma	Not revealed	Allogeneic T cells	Universal dual specificity CD19 and CD20 or CD22 CAR-T Cells	1/2	China	
NCT04557436	B-cell acute lymphoblastic leukemia	CD52 and TRAC	Allogeneic T cells	PBLTT52CAR19 gene therapy	1	United Kingdom	(45)
EUCTR2019-003462-40-GB	B-cell acute lymphoblastic leukemia	TCR and CD52	Allogeneic T cells	CAR19 + TCRαβ- T cells	1	United Kingdom	
NCT05631912	Non-Hodgkin lymphoma, B-cell	TRAC	Autologous T cells	Autologous CD19-STAR-T cell; phase 1 dose escalation (3 + 3): dose 1 (1 × 10^6 cells/kg), dose 2 (3 × 10^6 cells/kg), and dose 3 (1 × 10^7 cells/kg)	1/2	China	
NCT06014073	Non-Hodgkin lymphoma	TRAC and Power3	Allogeneic T cells	TRAC and Power3 genes knockout allogeneic CD19-targeting CAR-T cell (ATHENA CAR-T)	1/2	China	
NCT04213469	Relapse/refractory B-cell lymphoma	PD-1	Autologous T cells	Gene editing of autologous T cells with anti-CD19 ScFv expression and knockout of PD-1	1	China	(46)
NCT04767308	Relapsed/refractory hematopoietic malignancies	CD5	Autologous T cells	The endogenous CD5 in CT125A cells is knocked out via CRISPR/Cas9 genome editing technology to prevent fratricide during CAR-T cells manufacturing	Early 1	China	(47)
NCT05722418	Relapsed/refractory multiple myeloma	B2M and insertion of a B2M–HLA-E fusion protein	Allogeneic T cells	CB-011: an allogeneic CAR-T cell therapy targeting BCMA; cyclophosphamide chemotherapy for lymphodepletion and fludarabine chemotherapy for lymphodepletion	1	United States	
NCT04244656	Relapsed/refractory multiple myeloma	TRAC and B2M	Allogeneic T cells	CTX120: BCMA-directed T-cell immunotherapy comprised of allogeneic T cells genetically modified *ex vivo* using CRISPR-Cas9 gene editing components	1	United States Australia Canada, Spain	(48)
NCT04502446	T-cell lymphoma	CD70, TRAC and B2M	Allogeneic T cells	CTX130: CD70-directed T-cell immunotherapy comprised of allogeneic T cells genetically modified *ex vivo* using CRISPR-Cas9 gene editing components	1	United States Australia Canada	(49)
NCT05397184	Relapsed/refractory T-cell acute lymphoid leukemia	CD52, CD7 and TCR	Allogeneic T cells	Cryopreserved BE-CAR7 T cells (BE752TBCCLCAR7PBL); single-dose intravenous infusion (weight-based dosing) of a banded dose of CAR7+ T cells/kg BE-CAR7	1	United Kingdom	
ISRCTN15323014	T-cell acute lymphoid leukaemia	TRBC, CD7, and CD52	Allogeneic T cells	Single-dose intravenous infusion (weight-based dosing) of a banded dose of CAR7+ T cells/kg BE-CAR7	1	United Kingdom	(50)
NCT03545815	Mesothelin positive solid tumors	PD-1 and TCR	Autologous T cells	Anti-mesothelin CAR-T cells with PD-1 and TCR knockout	1	China	(51)
NCT03747965	Mesothelin positive solid tumors	PD-1	T cells	Mesothelin-directed CAR-T cells will be infused one day after the completion of conditioning regimen of paclitaxel and cyclophosphamide	1	China	
NCT04976218	Advanced EGFR positive solid tumors	TGFβR2	T cells	TGFβR-KO CAR-EGFR T cells	1	China	
NCT05812326	Advanced breast cancer	PD-1	Autologous T cells	PD-1 knockout anti-MUC1 CAR-T cells	1/2	China	
NCT04438083	Renal cell carcinoma	CD70, TRAC and B2M	Allogeneic T cells	CTX130: CD70-directed T-cell immunotherapy comprised of allogeneic T cells genetically modified *ex vivo* using CRISPR-Cas9 gene editing components	1	United States Australia Canada Netherlands	(52)
NCT05795595	Relapsed or refractory solid tumors	CD70, TRAC, B2M, Regnase-1 and TGFβR2	Allogeneic T cells	CTX131: CTX130 + disruption of Regnase-1 and TGFβR2 via CRISPR/Cas9	1/2	United States	(43)

TGFβR2, transforming growth factor β receptor 2; TRAC, TCR α constant; TRBC, TCR β constant; B2M, β2-microglobulin; HPK1, hematopoietic progenitor kinase 1; BCMA, B cell maturation antigen; EUCTR, EU Clinical Trials Register; ISRCTN, International Standard Randomised Controlled Trial Number.

Currently, the most successful and widely used CAR is the CD19-CAR, which recognizes the surface expression of CD19 on B lymphocytes. In a clinical trial for CD19^+^ relapsed or refractory lymphoma, autologous CD19-directing CAR-T therapy showed a significant durable tumor remission (86%–89%) during a median follow-up of 28.6 months in patients who had an initial response (55). Although CAR-T cell therapy has achieved remarkable results in the treatment of B-cell lymphoblastic leukemia, its application in treating other hematological and solid tumors has been less effective (56). One way to circumvent these hurdles is by combining CAR-T cell therapy with immune checkpoint blockade, such as target inhibition of programmed cell death protein 1 (PD-1; encoded by the *PDCD1* gene) and cytokine-inducible SH2-containing protein (CIS; encoded by the *CISH* gene) (57, 58).

PD-1 is present on the surface of activated T cells and regulatory T cells, and its ligand, programmed death-ligand 1 (PD-L1), is expressed by most cell types, including dendritic cells and tumor cells (59). The binding of PD-1 to its ligand mediates immune tolerance, inhibits T cell activation, and prevents tumor destruction (60). Functional disruption of the PD-1 locus by sgRNA:Cas9 led to upregulation of IFN-γ and enhanced primary human T cell cytotoxicity on tumor cell lines (61) and in patients with refractory NSCLC (62). Zhang et al. developed an enhanced type of CAR-T cells by integrating an anti-CD19 CAR cassette into the *PDCD1* gene locus through CRISPR/Cas9 (46). The modified CAR-T cells with attenuated PD1 expression exhibited higher proliferation capacity and eliminated tumor cells more rigorously than control CAR-T cells in mouse models. In a phase 1 clinical trial (NCT04213469), this CAR-T product was infused to treat patients with relapsed or refractory B-cell non-Hodgkin lymphoma and was highly effective even at a low infusion dose with a low percentage of CAR^+^ cells. During the 12 months follow-up study, complete (87.5%) and partial (12.5%) remission were observed in 8 patients without serious adverse events (46).

CRISPR-based techniques have also been used for the following genome editing in CAR-T cell therapies undergoing clinical trials (Table 1): (1) specific integration of the CAR cassette into the *AAVS1* safe harbor locus, or TCR alpha constant (TRAC) locus to enable CAR expression under the control of endogenous T cell promoter (46, 48); (2) knockout of the TCR in donor CAR-T cells to reduce the risk of graft versus host disease (GvHD) (45, 50); however, several studies indicated that endogenous TCR is essential for CAR-T cell long-term persistence *in vivo* (51, 63); (3) MHC I (the class I MHC) knockout by β2-microglobulin (*B2M*) disruption in donor CAR-T cells to avoid rejection of the CAR-T product by the patient’s own T cells (42, 49); and (4) knockout of certain T cell surface protein (e.g., CD5, CD7 and CD70) that is shared by tumor cells to eliminate CAR-T cell fratricide (killing each other) and increase its potency (43, 47, 50, 52). Through these modifications, especially with the elimination of MHC I and TCR molecules, universal CAR-T cells can be generated from healthy donors that have the potential for fast and cost-effective manufacturing (64). Of note, the transplanted MHC I-deficient CAR-T cells might be destroyed by natural killer cells due to the “missing self” response (65). One way to prevent this is to insert HLA-E while deleting HLA-A, B, and C, and thereby engineer cells that express HLA-E as their only surface MHC I molecule (66). These HLA-engineered cells are resistant to the cytotoxicity of natural killer cells and are not recognized as allogeneic by host CD8^+^ T cells; hence, this strategy is explored in CAR-T clinical trials (Table 1). Even though CRISPR-edited CAR-T cell therapies have shown improved therapeutic potential, serious adverse events have also been reported, such as cytokine release syndrome, lymphocyte and neutrophil cytopenia, and opportunistic infections, indicating careful risk evaluation of immunotherapy-related complications is necessary (50).

#### 3.1.2. Solid tumors

CRISPR-engineered CAR-T, TCR-T, and TIL therapies have been carried out in clinical studies to treat solid tumors, aiming to assess the safety and efficacy of these treatments. The application of CAR-T cell therapy in treating solid tumors remains a huge challenge due to limited infiltration and persistence, tumor microenvironment (TME) suppression, and the presence of suppressive T-cell regulatory mechanisms (67, 68). To overcome these barriers, CAR-T cells have been modified to disrupt one or multiple gene targets, including PD-1 (to reactivate T cells), TRAC (to prevent GvHD), B2M (to reduce T-cell-mediated rejection), CD70 (to prevent fratricide), Regnase-1 (to increase cellular expansion potential), and TGFβR2 (to avoid TME suppression) (Table 1). Preliminary results from these clinical trials indicate that CRISPR/Cas9-mediated gene disruption in CAR-T cells is relatively safe with no detectable off-target effects (51), but episodes of cytokine release syndrome were observed (52). In one clinical trial to treat renal cell carcinoma (NCT04438083), CAR-T cell therapy combined with disruption of CD70, TRAC, and B2M resulted in durable complete remission in one patient (7.7%) and stable disease in nine patients (69.2%) (52). Additionally, Regnase-1 and TGFβR2 knockouts added to the above edit could further improve the anti-tumor activity of CAR-T cells (43).

One limitation of CAR-T cell therapy is that CARs can only recognize surface antigens expressed on target cells, whereas TCRs recognize both cell surface and intracellular antigens that have been processed and presented as peptide-MHC molecules. These targets include tumor-associated antigens, cancer germline antigens, and tumor-specific neoantigens that are largely sequestered in the cytoplasm and nucleus of tumor cells (69). To enhance T cell recognition of tumor-associated self-antigens, genetically engineered T cells can be generated to express affinity-enhanced TCRs recognizing known tumor target antigens (69). Moreover, as TCRs are naturally developed, they can recognize epitopes at far lower concentrations than required for CAR-T cell activation. Therefore, TCR-T cell therapy shows greater prospects for treating solid tumors even though it is restricted by MHC molecules. To overcome TME inhibition, TCR-T cells are also engineered to block immune checkpoints. For example, there are four clinical trials based on TCR-T therapy combined with knockout of PD-1 via CRISPR/Cas9 to treat solid tumors (NCT04417764, NCT03081715, NCT02793856, and NCT03044743). In one trial to treat advanced NSCLC (NCT02793856), plasmids encoding Cas9 and sgRNA were electroporated into autologous primary T cells to disrupt genomic PD-1 expression. The edited T cells, with a median gene editing efficiency of 16%, were expanded *ex vivo* and re-infused as therapeutic T cells; of the 12 enrolled participants who had failed multiple lines of therapy before, the median progression-free and overall survival were 7.7 weeks and 42.6 weeks, respectively, with limited off-target effects, indicating the feasibility of clinical application of CRISPR/Cas9 gene-edited T cells (62).

TILs are naturally occurring heterogeneous groups of lymphocytes infiltrating solid tumors that can be isolated from the tumor site and expanded *ex vivo* by adding the T cell growth factor interleukin-2 (IL-2). The first TIL therapy in humans resulted in the regression of cancer in 60% of patients with metastatic melanoma (70). Compared with other adoptive cell therapies, TIL therapy exhibits diverse TCR clonality to recognize heterogeneous tumor antigens, superior homing ability to tumor sites, and low off-target side effects, holding great potential in combating solid tumors (71). TIL therapy combined with immune checkpoint blockade remains one of the most effective strategies to improve the efficacy of TIL. Disruption of the *PDCD1* or *CISH* gene has been actively investigated and proved useful in basic and preclinical studies (72, 73). To date, two clinical trials using *CISH*-inactivated TILs via CRISPR/Cas9 for the treatment of NSCLC and metastatic gastrointestinal cancers are underway (NCT05566223 and NCT04426669).

Above all, each form of ACT has its advantages and disadvantages; therefore, comprehensive considerations are required to select the most appropriate and effective treatment for different tumors.

### 3.2. Hematologic diseases

The inherited disorders of hemoglobin are among the most common monogenic diseases worldwide (74). According to the World Health Organization (WHO), SCD is more prevalent worldwide, while the thalassemic syndromes including α and β-thalassemia are associated with high prevalence rates in the WHO South-East Asia region. Currently, CRISPR/Cas9 (or Cas12)-based clinical trials in the blood system are mainly focused on β-thalassemia and SCD for interventional studies. The notable targeted genes, interventional methods, and trial conditions are summarized in Table 2.

**Table 2:** CRISPR-based clinical trials for β-thalassemia and SCD.

Disease	Trial ID	Disease condition	Target gene	Intervention	Gene editing method	Target size	Sponsor	References
β-thalassemia	NCT04211480	β-thalassemia major (β^0^/β^0^, β^+^/β^0^, β^E^/β^0^)	*BCL11A*	gene disruption	CRISPR/Cas9	6	BRL Medicine	(75)
	NCT05356195	TDT-homozygous or compound heterozygous β-thalassemia including β-thalassemia/hemoglobin E	*BCL11A*	gene disruption CTX001	CRISPR/Cas9	12	Vertex Pharmaceuticals	
	NCT03655678	TDT-same as above	*BCL11A*	gene disruption CTX001	CRISPR/Cas9	45	Vertex Pharmaceuticals	(76)
	NCT05577312	β-thalassemia major (β^0^/β^0^, β^+^/β^+^, β^+^/β^0^, β^E^/β^0^)	*BCL11A*	gene disruption BRL-101	CRISPR/Cas9	9	BRL Medicine	
	NCT04925206	TDT defined by protocol	*BCL11A*	gene disruption ET-01	CRISPR/Cas9	8	EdiGene (GuangZhou)	
	NCT04390971	TDT defined by protocol	*BCL11A*	gene disruption ET-01	CRISPR/Cas9	6	Institute of Hematology & Blood Diseases Hospital, China	
	NCT06041620	TDT defined by protocol	*HBG1/2* promoter	gene disruption VGB-Ex01	CRISPR/Cas12b	2	Institute of Hematology & Blood Diseases Hospital	
	NCT05444894	TDT-homozygous or compound heterozygous β-thalassemia including β-thalassemia/hemoglobin E	*HBG1/2* promoter	gene disruption EDIT-301	CRISPR/Cas12a	6	Editas Medicine	
	ChiCTR2100052858	TDT (β^0^/β^0^, β^+^/β^0^, β^E^/β^0^, β^+^/β^+^)	*HBG1/2* promoter	gene disruption RM-001	CRISPR/Cas9	10	Guangzhou Reforgene Medicine	(77)
	ChiCTR2100053406	TDT (genotype not indicated)	*HBG1/2* promoter	gene disruption RM-001	CRISPR/Cas9	5	Guangzhou Reforgene Medicine	(78)
SCD	NCT03745287	severe SCD as defined	*BCL11A*	gene disruption CTX001	CRISPR/Cas9	45	Vertex Pharmaceuticals	(76)
	NCT05329649	severe SCD as defined	*BCL11A*	gene disruption CTX001	CRISPR/Cas9	12	Vertex Pharmaceuticals	
	NCT04443907	SCD (β^S^/β^S^, β^S^/β^C^, β^S^/β^0^ or others)	*BCL11A*	gene disruption OTQ923	CRISPR/Cas9	20	Novartis Pharmaceuticals	
	NCT05951205	β^S^/β^C^	*BCL11A*	gene disruption Exa-cel	CRISPR/Cas9	12	Vertex Pharmaceuticals	
	NCT04774536	severe SCD as defined	*HBB*	gene correction CRISPR-SCD001	CRISPR/Cas9	9	Mark Walters, MD-UCSF	
	NCT05456880	SCD with β^S^/β^S^, β^S^/β^0^, or β^S^/β^+^ genotypes & Severe SCD as defined	*HBG1/2* promoter	gene correction BEAM-101: an ABE	Base editing	15	Beam Therapeutics	
	NCT04853576	severe SCD (β^S^/β^S^, β^S^/β^0^, β^S^/β^+^)	*HBG1/2* promoter	gene disruption EDIT-301	CRISPR/Cas12a	40	Editas Medicine	
β-thalassemia & SCD	NCT05477563	TDT- same as above; severe SCD	*BCL11A*	gene disruption CTX001	CRISPR/Cas9	12	Vertex Pharmaceuticals	

#### 3.2.1. β-Thalassemia

β-thalassemia is an autosomal recessive disorder caused by various mutations (i.e., point mutations, insertions, and deletions) in the human β-globin gene (*HBB*), and the severe forms of β-thalassemia media or major could be life-threatening, characterized by the reduction (β^+^) or absence (β^0^) of β-hemoglobin chains (79–81). Hemoglobin A (HbA, α_2_β_2_), the adult hemoglobin, is composed of two α-and two β-hemoglobin chains. Hence, the decrease of β-hemoglobin chains can lead to an imbalance of the α/β globin ratio and precipitation of free α-hemoglobin, which can damage the cell membrane of erythrocytes, causing anemia and large invalid erythropoiesis (80). β-thalassemia major is the most severe form with a homozygous β^0^/β^0^, β^+^/β^+^, or heterozygous β^0^/β^+^, β^E^/β^0^ genotype (81). Among them, β^E^ (hemoglobin E) is a β-hemoglobin variant with a point mutation in the *HBB* gene (HBB:c.79G > A), producing slightly decreased β-globin and manifesting mild clinical symptoms (82). However, coinheritance of β^E^ with β^0^ mutation causes the most common severe form of thalassemia in Southeast Asia (82). Both β-thalassemia major and media (β^0^/β^+^ or β^+^/β^+^) are transfusion-dependent β-thalassemia (TDT), but frequent blood transfusion can lead to iron overload and metabolic disorders that can be fatal; thereby, iron chelation therapy needs to be performed simultaneously to mitigate those side effects (79). Overall, these conventional therapies are inconvenient, costly, and incurable.

CRISPR/Cas9 and its expanded technologies, such as base editing and prime editing, have been extensively explored and shown promise to cure β-thalassemia in basic research and pre-clinical studies (83–85). The genome-editing strategies include (1) correction of the causative mutations of the *HBB* gene via HDR (83, 86); (2) inhibition of α-globin gene (*HBA*) expression to balance the α/β globin ratio (87, 88); and (3) induction of fetal hemoglobin (HbF, α_2_γ_2_) expression in β-thalassemia patient-derived hematopoietic stem and progenitor cells (HSPCs) or hematopoietic stem cells (HSCs) (89, 90). Human HbF is the major hemoglobin in fetal red blood cells (RBCs) and is almost completely replaced by HbA within 1 year of age due to a shift from γ-globin gene (*HBG*) to *HBB* gene expression (91). Hereditary persistence of fetal hemoglobin (HPFH) is a naturally occurring benign condition mostly caused by point mutations and deletions in the β-globin cluster that repress the γ-globin to β-globin switch, resulting in the elevation of HbF in adult erythrocytes (92). Accumulated evidence has shown that coinheritance of β-thalassemia with HPFH presents mild symptoms, implying that β-thalassemia can be alleviated by promoting HbF resurgence (92). Generally, there are two ways to increase *HBG* expression. One is disrupting B-cell lymphoma/leukemia 11A (BCL11A), an *HBG* transcriptional repressor, by targeting its erythroid enhancer at the +58 site (+58 kb from the transcription start site of the *BCL11A* gene) that binds to the transcription factor GATA1 to enhance BCL11A expression (89). The other way is to disrupt the BCL11A binding site in the *HBG1/2* promoter (115 bp upstream of the transcription start site) to mimic HPFH (90). Both strategies have been validated as efficient for HbF reproduction via CRISPR/Cas9-mediated NHEJ (89, 90).

In current CRISPR clinical trials for TDT (β^0^/β^0^, β^+^/β^0^, β^E^/β^0^, and β^+^/β^+^) treatment, nearly all interventional studies are designed to disrupt the erythroid-specific enhancer of the *BCL11A* gene (7 studies) or the *HBG1/2* promoter (4 studies) to reactivate γ-globin expression in autologous HSPCs or HSCs (Table 2). There is only one trial of unknown status (NCT03728322) aimed at investigating the safety and efficacy of the *HBB* gene correction in TDT patient-specific iHSCs using CRISPR/Cas9. In a groundbreaking clinical study (NCT03655678), CRISPR/Cas9-based editing of the *BCL11A* erythroid-specific enhancer was carried out to induce HbF resynthesis in CD34^+^ HSPCs (CTX001) from a β^0^/β^+^ patient (76). The allelic editing frequency of CTX001 was 68.9% *ex vivo*. After myeloablation, CTX001 was intravenously transplanted back into the patient with allelic editing frequency attaining 62.9% in nucleated peripheral blood cells in month 18 and 76.1% in CD34^+^ cells of the bone marrow in month 12. The HbF level of the patient increased from 0.3 g/dL at baseline to 13.1 g/dL in month 18, as did F-cells (the proportion of circulating erythrocytes expressing HbF), which increased from 10.1% at baseline to 99.7% in month 6, and the patient became transfusion-independent. During the 21.5 months follow-up, the patient had 32 adverse events, most of which were classified as grade 1/2 in severity except for pneumonia in the presence of neutropenia and veno-occlusive liver disease with sinusoidal obstruction syndrome (VOD-SOS). Nevertheless, all these adverse events were resolved after treatment. In summary, even if *ex vivo* editing of CD34^+^ HSPCs decreases their stemness, CTX001 still “homes” to the bone marrow to exert its therapeutic function (76). Similarly, Fu et al. (NCT04211480) also successfully targeted the GATA1-binding site at the +58 *BCL11A* erythroid enhancer in CD34^+^ HSPCs of two children (β^0^/β^0^ and β^+^/β^+^) with no obvious side effects, and both patients achieved transfusion independence for more than 18 months with over 85% editing persistence in bone marrow cells (75). In addition, Wang et al. conducted an initial safety and efficacy study (ChiCTR2100052858) for autologous infusion of the HSPCs with disrupted *BCL11A* binding site on the *HBG1/2* promoter (RM-001) in two β^0^/β^0^ patients, and both demonstrated successful engraftment without severe adverse effects (77). At 3 months post-gene-edited HSPCs infusion, both patients had been transfusion-free, with total Hb and HbF levels reaching 11 g/dL and 9.7–9.8 g/dL, respectively, and approximately 89% of peripheral erythrocytes being F-cells (77). In the most recent update, another 4 patients with either β^0^/β^0^ or β^0^/β^+^ genotype also achieved transfusion independence (total Hb continued ≥9 g/dL) after RM-001 infusion (78).

#### 3.2.2. Sickle cell disease

SCD is an autosomal recessive disease caused by a single point mutation at the sixth codon of the *HBB* gene (HBB:c.20A > T), resulting in the substitution of hydrophilic glutamic acid to hydrophobic valine and sickle hemoglobin (HbS, β^S^) formation. HbS is prone to polymerization upon deoxygenation and forms long-chain polymers that distort the shape of RBCs, making them fragile, rigid, and unable to deform when passing through narrow capillaries, leading to vessel occlusion and hemolysis (93). SCD has quite variable clinical manifestations, and the homozygous state for HbS (β^S^/β^S^) is the most common and severe type, called sickle cell anemia (94).

The coinheritance of HPFH and SCD can mitigate SCD symptoms (95). Therefore, similar to β-thalassemia, genome editing therapies for SCD include not only *HBB* mutant gene correction via HDR but also gene disruption of *BCL11A* or *HBG1/2* promoters to reactivate *HBG* expression via NHEJ (90, 96) (Table 2). In Frangoul’s study, a SCD patient infused with CTX001 achieved an average allelic editing frequency of 80%; the HbF and HbS levels changed from 9.1 and 74.1% at baseline to 37.2 and 32.6% after 3 months treatment; F-cell expression was maintained at nearly 100% through month 15; and the patient became transfusion-free (76). Meanwhile, this patient was identified with 114 adverse events during 16.6 months after CTX001 infusion, and three of them were categorized as severe adverse events that were resolved after treatment: sepsis in the presence of neutropenia, cholelithiasis, and abdominal pain (76). Even though there is no evidence of CTX001 off-target editing *in vitro*, *in vivo* off-target analysis needs to be executed on clinical samples.

With the evolution of CRISPR/Cas technology, base editors emerge as novel sgRNA-guided gene editing platforms that can make precise single base changes without cutting the DNA double helix. The phase 1/2 clinical trial NCT05456880 led by Beam Therapeutics Inc. aimed to generate an A to G base swap in the *HBG1/2* promoters via base editing to mimic HPFH in severe SCD patients and represented the first clinical trial of a base editor in the United States. In addition, a new approach for correction of the HbS sickle mutation (HBB:c.20A > T) by ABE (T-to-C transition) is under development by this company, leading to the production of a naturally occurring benign hemoglobin G-Makassar variant compatible with normal hemoglobin function (97, 98). With the development of adenine transversion base editor AYBE that makes T-to-A transversion possible (35) and prime editors that can introduce all possible base-to-base conversions, correction of the SCD causative mutation to wild-type *HBB* can be achieved, showing promise to cure SCD (24).

Collectively, CRISPR/Cas9 and its newly evolved base editing and prime editing technologies are promising genome editing tools to cure hereditary blood disorders, not only because they enable site-specific modifications of the human genome for targeted gene editing but also because the edited HSPCs can home to their bone marrow niches, self-renew, and provide durable therapeutic benefits.

### 3.3. Respiratory system diseases

In the respiratory system, 5 out of 8 CRISPR-based clinical trials are related to the coronavirus disease-2019 (COVID-19), which is caused by the severe acute respiratory syndrome coronavirus 2 (SARS-CoV-2) and has produced a global pandemic in the past 3 years (99). Due to the strong infectivity and high variability of COVID-19, how to effectively control the spread of the virus and reduce the infection and mortality rate were extremely urgent and important at that time. Therefore, developing rapid, reliable, economical, and convenient diagnostic tests was essential for the timely detection of SARS-CoV-2 infection and subsequent transmission control.

Real-time reverse transcription PCR (rRT-PCR) for detecting the genomic RNA of SARS-CoV-2 is considered the gold standard for diagnosing COVID-19 because of its high sensitivity and specificity (100). The target sequences amplified include those encoding the structural proteins, namely the spike (S), nucleocapsid (N), membrane (M), and envelope (E), as well as those encoding the non-structural RNA-dependent RNA polymerase (RdRp) and replicase open reading frame 1a/b (ORF1a/b) (101). However, this technique requires expensive equipment and trained personnel to conduct the test, and the turnaround time could be several days. In addition, the lateral flow rapid test is a rapid antigen test that usually detects the S and N antigens of SARS-CoV-2, can be completed within 20 minutes, and does not need laboratory processing. However, this method is much less sensitive as compared to rRT-PCR, and a low viral load may lead to false negatives. Thus, it is usually used as an initial screening test (102).

**Figure 4:**
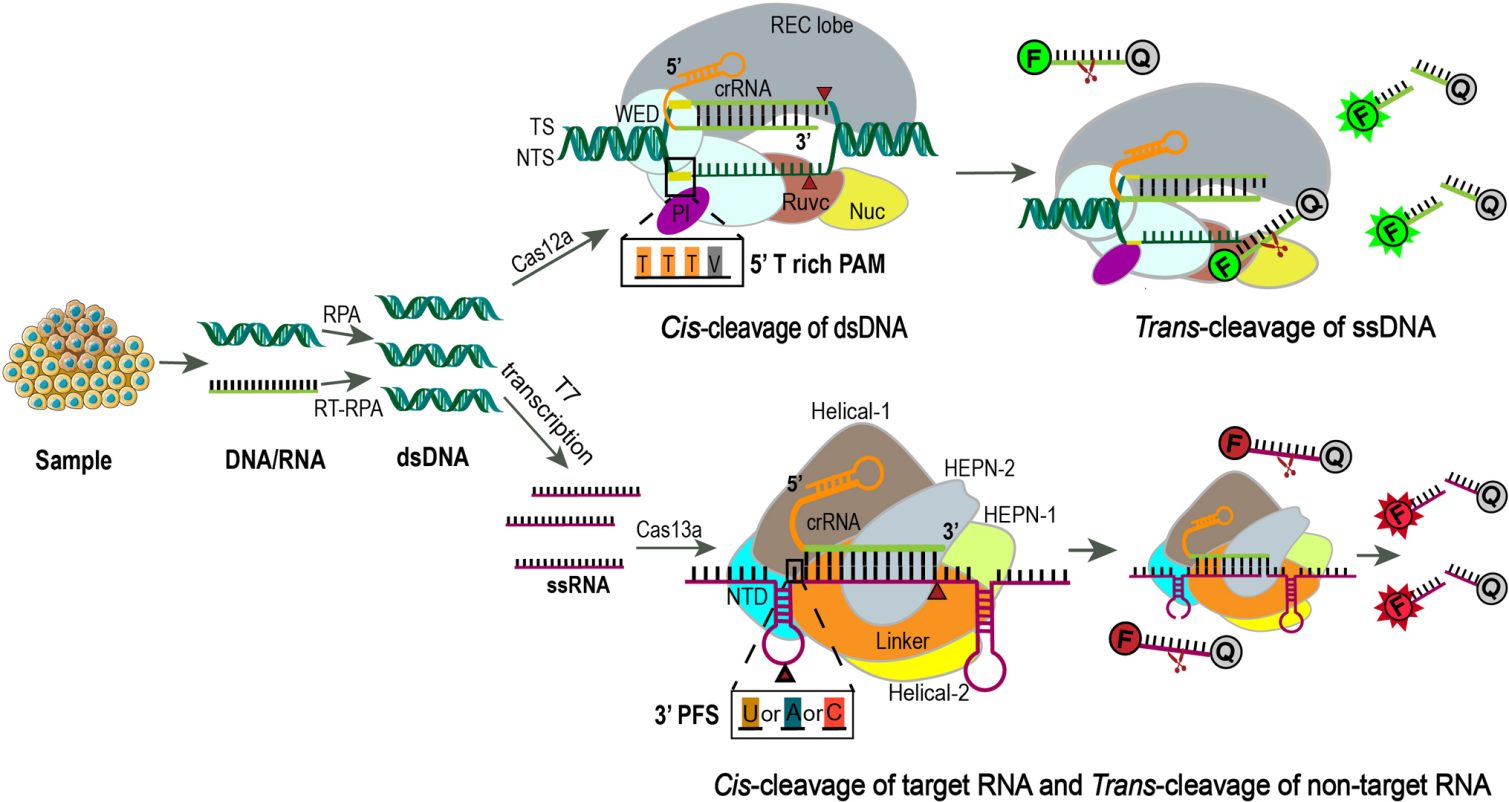
Using CRISPR-Cas12a or Cas13a for precision diagnostics. Samples are extracted for DNA or RNA and amplified using RPA or RT-RPA, respectively. For Cas13a detection, the RPA products are further transcribed to RNA by T7 RNA polymerase. Then the amplified nucleotides are combined with Cas12a or Cas13a, crRNA, and an inactivated fluorescent ssDNA or ssRNA reporter. Upon crRNA binding and cleavage of the specific targeting sequence, the collateral activity of Cas12a or Cas13a will enable the cleavage of the inactivated reporter, resulting in activation of the fluorophore as an indicator for the presence of the target sequence (111, 112). Cas12a is an endonuclease with bilobed architecture, containing a recognition (REC) and a nuclease (NUC) lobe, the latter one harboring the wedge (WED), PAM-interacting (PI), bridge helix (BH), RuvC, and Nuc domains (113, 114). Binding of a single crRNA with the target strand (TS) induces conformational change and exposure of the catalytic site in the RuvC domain that cleaves the non-target strand (NTS) at 18 bp and TS at 23 bp downstream of the 5’-TTTV-3′ (V = A/G/C) PAM sequence sequentially to generate sticky ends (114). Subsequently, the release of the PAM-distal cleaved DNA keeps Cas12a catalytically active and allows collateral ssDNA *trans*-cleavage (115). F, fluorescence; Q, quencher. Cas13a is a single crRNA-guided RNase mediating ssRNA cleavage, consisting of a REC lobe and a NUC lobe. The REC lobe contains an N-terminal domain (NTD) and a Helical-1 domain. The NUC lobe contains two higher eukaryotic and prokaryotic nuclease (HEPN) domains, a linker that connects these two domains, and a Helical-2 domain (116). When the target ssRNA and crRNA are combined to form double-stranded RNA, the conformational change of the HEPN domains activates Cas13a, which relies on the protospacer flanking sequence (PFS, 3’ U, A, or C) to efficiently cut the targeted ssRNA and then non-specifically cut any ssRNA nearby (29).

CRISPR/Cas systems have evolved as next-generation molecular diagnostic platforms for fast, sensitive, accurate, and cost-effective detection of nucleic acids (103). As for SARS-CoV-2 detection, four clinical trials (NCT05107258, NCT05331976, CTRI/2021/02/030950, and ChiCTR2000029810) were carried out to evaluate CRISPR/Cas12a or CRISPR/Cas13a-based assays, which generally include four steps: sample pretreatment (including pre-amplification), CRISPR/Cas-based target recognition of SARS-CoV-2 sequences, concomitant cleavage of reporter molecules, and signal detection (104). Briefly, viral RNA is extracted from nasopharyngeal or oropharyngeal swabs, reverse transcribed, and amplified into dsDNA by reverse transcription-recombinase polymerase amplification (RT-RPA). RPA is an isothermal amplification alternative to PCR that utilizes a recombinase, a single-stranded DNA binding protein, a strand-displacing DNA polymerase, and two opposing primers to amplify sequence-specific nucleic acids (105, 106). The advantages of RPA are obvious, as it can be performed at a constant temperature (usually 37–42°C) without a thermal cycler, and the reaction processes quickly in 10 minutes, making it particularly suited for point-of-care diagnostics, especially in resource-constrained environments (106). In the second step, the CRISPR/Cas system can specifically recognize the amplified viral target sequence under the guidance of gRNA, which specifically binds to SARS-CoV-2. The two most frequently used Cas proteins are Cas12a and Cas13a, which are fundamental elements in DETECTR and SHERLOCK assays, respectively (29, 107, 108). In addition to snipping targeted dsDNA by Cas12a or single-strand RNA (ssRNA) by Cas13a, Cas12a and Cas13a both display non-specific *trans* cleavage of single-stranded nucleic acids nearby, an effect dubbed “collateral activity.” The difference between the two is that Cas12a cleaves ssDNA, whereas Cas13a cleaves ssRNA (109, 110). By adding fluorescein dye-quencher reporters (ssDNA or ssRNA) to the CRISPR/Cas12a or Cas13a system, the cleavage of the quenching group will release the fluorescent group and let it shine, indicating detection of target sequence (107) (Figure 4). The main objective of these clinical studies is to determine the sensitivity and specificity of the CRISPR SARS-CoV-2 tests compared to conventional rRT-PCR, but so far there are no published results derived from these clinical trials.

Based on the same principle of SARS-CoV-2 detection, CRISPR/Cas12a and Cas13a-assisted nucleic acid detection are also employed for the diagnosis of other pulmonary diseases, such as pneumonia and *Mycobacterium tuberculosis* (NCT05143593, NCT04178382, and NCT04074369). In one study, Wang et al. showed that using CRISPR/Cas12a technology with bacterial species-specific DNA tags can achieve rapid bacteria strain detection in 4 h with 100% sensitivity and over 87% specificity in pneumonia patients, ensuing efficient antibiotic selection and therapeutic intervention (117). In summary, CRISPR-based technologies provide a sensitive, specific, and convenient tool with low cost and minimum instrument requirements for molecular diagnostics.

### 3.4. Endocrine, nutritional, or metabolic system disorders

In this category, we screened out a total of seven clinical trials, of which the following are representative.

#### 3.4.1. Type 1 diabetes

Type 1 diabetes mellitus is an insulin-dependent diabetes caused by autoimmune destruction of pancreatic islet β cells, and human pluripotent stem cell (hPSC)-derived β cell replacement therapies are effective yet hindered by immune rejection of grafted cells and the potential recurrence of autoimmunity to disrupt long-term success (118, 119). Several strategies have been applied to circumvent this problem, such as utilizing immunosuppression drugs, inducing immune tolerance, and encapsulating transplanted cells in an immunoprotective device that allows diffusion of small molecules, namely glucose, insulin, and oxygen, but prevents infiltration of immune cells (120). Alternatively, CRISPR/Cas9-based multiplex genome editing can enable hPSCs or human embryonic stem cells to evade host adaptive and innate immune rejection upon cell engraftment, and the editing targets include HLAs, PD-L1, chemokine ligand 10 (CXCL10), and so on (121–124). For example, hypoimmunogenic cell grafts can be generated for allogeneic transplantation by disrupting *B2M* and class II transactivator (*CIITA*) genes, the major components of HLA class I and class II, respectively, in induced pluripotent stem cells (iPSCs), while overexpressing CD47 to avoid phagocytosis by the host’s innate immune system (125). These gene-edited stem cells retain their pluripotency, can be differentiated into functional pancreatic β cells, and exhibit long-term survival *in vivo* without any immunosuppression (122, 124, 125). Currently, two clinical trials (NCT05210530 and NCT05565248) sponsored by CRISPR Therapeutics and ViaCyte are being launched to evaluate the safety and tolerance of the gene-edited cell replacement therapy in type I diabetic subjects, but the exact gene editing targets are not revealed, and no results have been reported.

CRISPR/Cas9 gene editing has the potential to treat non-immune-mediated monogenetic forms of diabetes as well. Ma et al. used CRISPR/Cas9 and a single-strand correction template to target a point mutation at the start codon of the insulin gene (INS:c.3G > A) in patient iPSCs, and 3 out of 61 iPSC colonies showed the desired gene correction (126). The corrected iPSCs were induced to differentiate into pancreatic endocrine cells, which produced insulin at levels similar to wild-type controls and could maintain normal blood glucose homeostasis once grafted into an immune-compromised diabetic mouse model (126). Despite the occurrence of several off-target effects, this proof-of-principle study suggests the potential of autologous transplantation of gene-corrected stem cells for the treatment of monogenic diabetes.

#### 3.4.2. Heterozygous familial hypercholesterolemia (HeFH)

HeFH is an autosomal codominant disease characterized by an elevated plasma low-density lipoprotein-cholesterol (LDL-C) level that may lead to atherosclerosis and a heart attack if left untreated. HeFH is often caused by loss-of-function of the LDL receptor (*LDLR*) or apolipoprotein B (*APOB*) gene or gain-of-function of the proprotein convertase subtilisin/kexin type 9 (*PCSK9*) gene (127). ApoB on the LDL particle acts as a ligand for LDLRs expressed on the liver and other cell membranes; upon ligand-receptor binding, LDL particles carrying cholesterol and triglycerides are removed from the circulation, internalized, and delivered to lysosomes for degradation. PCSK9 binds to LDLR and targets it for lysosomal degradation instead of recycling it back to the plasma membrane, leading to decreased intake of LDL into cells and increased plasma LDL-C (127). Gain-of-function *PCSK9* variants have been identified as missense mutations in the coding region, intronic junctions, as well as in the promoter region that increase PCSK9 transcription and are associated with hypercholesterolemia and coronary heart disease (CHD) (128). In contrast, the loss-of-function *PCSK9* variants, such as nonsense mutations (Y142X and C679X), have demonstrated the opposite effects, showing a 40% reduction in plasma LDL-C levels and a reduced risk of CHD (129, 130). Hence, targeting PCSK9 has been a hot spot for HeFH therapy.

Currently, two monoclonal antibodies (Repatha and Praluent) against the PCSK9 protein have been approved by the FDA since 2015, and both agents need to be injected subcutaneously monthly or every 2 weeks. The clinical trial with an oral PCSK9 inhibitor demonstrated statistically significant and dose-dependent reductions of LDL-C up to 60.9% from baseline at week 8 (131). Alternatively, in an ongoing clinical trial for HeFH (NCT05398029), adenine base editor is delivered through lipid nanoparticles (LNPs) to make an A-to-G substitution at a specific site of the *PCSK9* gene *in vivo*, disrupting its gene expression to lower circulating PCSK9 and LDL-C levels. The safety and pharmacodynamic profile of this treatment are still under investigation. Since HeFH is the most common genetic disease in humans, with a prevalence of around 1:300 (132), gene editing therapies hold great potential to provide an economical “once-and-done” treatment that may confer an active long-term effect.

#### 3.4.3. Hereditary transthyretin amyloidosis

Transthyretin amyloidosis (ATTR) is a progressive, life-threatening multisystem disease caused by the misfolding of transthyretin (TTR), which is a homotetramer mainly produced in the liver. ATTR is classified as acquired form (wild-type ATTR) and inherited form (variant or hATTR); the latter one is an autosomal dominant disorder that can be caused by more than 100 types of *TTR* gene mutations (133, 134). These *TTR* gene mutations result in the dissociation of TTR tetramers into monomers and protein misfolding to produce insoluble toxic amyloid fibril aggregates in the extracellular space of various tissues, of which neural and myocardial deposition are most common, leading to ATTR polyneuropathy and ATTR cardiomyopathy, respectively (133).

Orthotopic liver transplantation (OLT) to replace variant TTR with donor-derived wild-type TTR is an optional treatment for hATTR (135). However, this treatment is limited by donor availability, the requirement for lifelong immunosuppression, and other side effects. In addition, small molecules that stabilize the TTR tetramer, antisense oligonucleotides or small interfering RNA that induce TTR mRNA degradation, and fibril disruptors are currently in different stages of clinical trials (133, 134).

CRISPR/Cas9 gene editing technology provides an alternative approach for the treatment of hATTR through *in vivo TTR* gene editing. Among the clinical trials we screened, one (NCT04601051) was a clinical evaluation of the *in vivo* gene editing therapeutic agent NTLA-2001, which used LNPs to encapsulate Cas9 mRNA and a sgRNA targeting the *TTR* gene (136). Through intravenous injection, NTLA-2001 was intended to knock out the *TTR* gene in hepatocytes since LNP is opsonized by apolipoprotein E upon entry into circulation, which can bind to LDL receptors on the hepatocyte plasma membrane and facilitate endocytosis of LNP. This targeted delivery to the liver can maximize efficacy and greatly reduce systemic toxicity. In this phase I clinical trial, six hATTR patients with polyneuropathy received NTLA-2001 injection, and 28 days later, circulating TTR levels were reduced in a dose-dependent manner, with an average of 52 and 87% TTR reduction in the lower-dose group and the higher-dose group, respectively (136). Moreover, no serious adverse events were found. As a circulating transport protein, TTR has physiological functions in thyroxine (137) and retinol (138) transport. Thyroid hormone thyroxine distribution is regulated by multiplex factors, and TTR is not essential for tissue uptake of thyroxine (139). On the other hand, circulating retinol and retinol-binding protein levels in TTR-knockout mice were significantly reduced to those seen in vitamin A-deficient animals that are near death (140, 141). Since NTLA-2001 can lead to decreased production of both wild-type and mutant TTR, vitamin A supplements were given to patients to avoid vitamin A deficiency. The authors chose this design instead of using a mutation-specific gene-editing approach for two reasons: (1) it has been shown that after OLT, wild-type TTR continues to deposit in the hearts of hATTR patients with cardiac amyloidosis, leading to poor outcomes (142); and (2) the current approach provides a potential universal solution for all hARRT patients despite mutation type. Even though encouraging, the long-term effects of CRISPR/Cas9-mediated genome editing should be carefully monitored, and the overall survival and phenotypic improvement after NTLA-2001 treatment should be assessed in the future.

### 3.5. Immune system diseases

Acquired immunodeficiency syndrome (AIDS) is a systemic disease caused by human immunodeficiency virus (HIV) infection, and HIV-1 is the most common type. HIV targets and destroys CD4^+^ T lymphocytes (helper T cells), which coordinate the immune response by stimulating other immune cells such as CD8^+^ lymphocytes and macrophages to fight infection. At present, antiretroviral therapy (ART) is the key to reducing the host viral load, but it can only inhibit the replication of the HIV-1 retrovirus instead of acting on the integrated HIV pro-viral DNA (reversely transcribed from HIV RNA) in the genome of infected CD4^+^ T cells (143). Hence, HIV pro-viral DNA can replicate, which serves as the basis for long-term viral latency and the source of viral rebound after drug withdrawal, and the patients must rely on the expensive ART treatment for life. Therefore, searching for therapies targeting the removal of HIV pro-viral DNA in the host genome is the focus of AIDS treatment (144).

Kaminski et al. reported an effective eradication of integrated HIV-1 DNA from latently infected human CD4^+^ T cells by lentiviral delivery of the Cas9/sgRNA complexes that target the HIV-1 long terminal repeat, achieving a reduction of viral copies and the prevention of new HIV-1 infection without causing genotoxicity to the host DNA (145). Subsequent *in vivo* studies demonstrated that intravenous adeno-associated virus (AAV) 9 delivery of Cas9 and dual sgRNAs into HIV-1-infected humanized mice achieved 60 to 80% efficiency of HIV-1 viral DNA excision, leading to the elimination of integrated pro-viral DNA from blood cells and other tissues known to be HIV reservoirs without off-target effects (146). Similar results were achieved in a non-human primate model of HIV infection (147). The success of these studies laid the foundation for human clinical trials to evaluate the safety and efficacy of a similar approach, EBT-101, in which the CRISPR/Cas9 system was delivered by AAV9 for intravenous administration to target HIV-1 pro-viral DNA in HIV-1-infected adults on stable ART (NCT05144386), and to assess the long-term safety of EBT-101 for 15 years (NCT05143307). The first participant receiving a single-dose intravenous infusion of EBT-101 is under observation and will soon be evaluated for viral rebound and eligibility for stopping ART under the protocol.

Entry of HIV-1 into host cells requires cell surface CD4 and additional coreceptors such as C-C chemokine receptor type 5 (CCR5) (148). Individuals with a naturally occurring homozygous 32-bp deletion (Δ32) of the *CCR5* gene showed a lack of CCR5 expression and were nearly completely resistant to HIV-1 infection (149). Therefore, CRISPR/Cas9 gene editing was used to create a similar CCR5-Δ32 mutation in human CD34^+^ HSPCs, which were transplanted to severely immunodeficient mice with sub-lethal irradiation and conferred HIV-1 resistance as indicated by a significant reduction of virus titer and enrichment of human CD4^+^ T lymphocytes (150). Later, the same research group initiated a clinical trial (NCT03164135) to evaluate the safety and feasibility of CRISPR-edited CCR5-ablated CD34^+^ HSPC allotransplantation into HIV-infected subjects with hematological malignancies. In a recruited AIDS patient with acute lymphoblastic leukemia, donor-derived HSPCs carrying the ablated CCR5 can differentiate into multiple hematopoietic lineages and persist for more than 19 months without gene editing-related adverse events, demonstrating the feasibility of long-term engraftment of gene-edited cells (151). In addition, after HSPC transplantation, the peripheral blood CD4^+^ T cells recovered to their normal range by month 6, and the acute lymphoblastic leukemia was in complete remission for 19 months, even though CCR5 editing efficiency in bone marrow cells was less than 8.28% (151). These results are promising, but more patients need to be evaluated, and a thorough assessment of the potential risks of CRISPR/Cas9-mediated CCR5 ablation in HSPCs under higher gene-targeting efficiency is warranted.

## 4. Conclusions and future perspectives

CRISPR/Cas gene editing technology has made remarkable progress since its discovery. Due to its ease of use and high targeting and editing efficiency, it has been widely used in clinical research on the treatment of tumors and monogenetic disorders, as well as sensitive and specific nucleic acid sequence detection. However, there are still many hurdles to overcome before this technique is broadly used in treating human diseases.

First, CRISPR/Cas-based genome editing is restricted by PAM recognition with specific Cas proteins. Therefore, the discovery or modification of Cas proteins with various PAM coverages is very important to advance this technology. The commonly used SpCas9 and SaCas9 recognize target sites with NGG (N = any nucleotide) PAMs (6) and NNGRRT (R = A or G) PAMs (1), respectively. Using phage-assisted evolution (152), Shannon et al. obtained three new SpCas9 variants that recognize NRRH, NRTH, and NRCH (H = A, C, or T) PAM sequences and enable targeting previously inaccessible genomic sequences with non-G PAMs (153). Moreover, these evolved variants exhibit similar or higher editing efficiencies than wild-type SpCas9, thus greatly expanding the targeting limitations of the CRISPR/Cas9 system to a certain extent. Meanwhile, with structure-guided engineering, Walton et al. generated SpCas9 variants that recognize NGN, NRN, and NYN (Y = C or T) PAMs, enabling broader Cas9 nuclease and base editing applications (154).

Secondly, the off-target effect generated by non-specific targeting of sgRNA to the genome can lead to adverse consequences such as gene mutations and oncogene activation in the genome and bring risks to clinical applications (155). Previous studies demonstrated that SpCas9 nucleases cause high-frequency off-target mutations in human cells with imperfectly matched RNA–DNA interfaces, especially with PAM-distal mismatches (if mismatches are distal to the PAM site) (156, 157). Through structure-guided design, “enhanced specificity” SpCas9 (eSpCas9) variants were generated with amino acid substitutions that weaken the interactions between Cas9 and the non-target strand, which can significantly reduce the off-target indel mutations and maintain a strong on-target cleavage (158). A hyper-accurate Cas9 variant (HypaCas9) was produced during the in-depth exploration of the SpCas9 proofreading mechanism (159). Wild-type SpCas9 has two catalytic domains: the HNH and RuvC endonuclease domains that cleave target and non-target strands, respectively. Upon binding to the target strand, the HNH domain undergoes conformational rearrangement, activating the RuvC nuclease domain to synergistically cleave both DNA strands (160). The HNH domain docks in an active state with on-target binding but is loosely trapped in a non-catalytic conformational checkpoint with mismatched target binding (161). In addition, the non-catalytic domain REC3 of Cas9 acts as an allosteric effector that binds to the sgRNA/DNA duplex and regulates HNH nuclease activation. HypaCas9 generated by targeted mutagenesis within the REC3 domain increases the threshold for HNH conformational activation upon binding to DNA substrates, i.e., more stringently traps the HNH domain in the conformational checkpoint in the presence of mismatches, thereby reducing off-target cleavage (159).

Thirdly, how to deliver the CRISPR/Cas system successfully and efficiently into the human body is still a major challenge for genome editing. The methods of delivery can be classified as viral- or non-viral-based, and the commonly used viral vectors include AAV, adenovirus, and lentivirus (162). Among them, AAV has become the most widely accepted viral vector for gene therapy *in vivo* due to its mild immunogenicity and good therapeutic effect in a broad range of animal models and clinical trials (163, 164). In addition, the AAV genome integrates specifically into the *AAVS1* site of human chromosome 19, a safe genomic location that can host a high level of integrated gene expression, avoiding random insertional mutagenesis (165). However, given the limited packaging capacity of AAVs (4.7 kb), the Cas9 gene (3.1–4.2 kb), sgRNA, and donor template have to be packed into different AAV vectors (166). Non-viral-based delivery methods include electroporation, lipid nanoparticles, hydrodynamic injection, etc., which are safe with a broader packaging capacity but have a low delivery efficiency (167, 168). A recent study demonstrated the possible application of an extracellular contraction injection system (eCIS): photorhabdus virulence cassette (PVC) for protein delivery in mammalian cells through specific interaction between the PVC tail fiber and the target cell (169). The engineered PVC can load the SpCas9 protein and mediate on-target gene editing in human cells. Furthermore, PVC-mediated protein delivery in live mice was not immunogenic or toxic, suggesting its potential use for human gene therapy (169). In general, the development of a safe delivery method that can meet the needs of clinical treatment has become a key issue in the application of CRISPR technology.

In summary, the existing limitations can be conquered with the in-depth exploration of CRISPR gene editing technology, and we anticipate its further advancement, wider translation, and improved effectiveness in various clinical settings in the near future.

## Author contributions

SZ: Writing – original draft, Writing – review & editing. YiW: Writing – original draft, Writing – review & editing. DM: Writing – original draft, Writing – review & editing. YuW: Writing – original draft, Writing – review & editing. HZ: Writing – original draft, Writing – review & editing. YP: Writing – original draft, Writing – review & editing. YzW: Writing – review & editing. ST: Supervision, Writing – review & editing. PH: Conceptualization, Funding acquisition, Supervision, Writing – review & editing.
